# The gut microbiota-neuroimmune crosstalk and neuropathic pain: a scoping review

**DOI:** 10.1017/gmb.2023.7

**Published:** 2023-06-19

**Authors:** Gayani Nawarathna, Kausar S. Fakhruddin, Ali I. S. A. Shorbagi, Lakshman P. Samaranayake

**Affiliations:** 1 Department of Basic Sciences, Faculty of Dental Sciences, University of Peradeniya, Peradeniya, Sri Lanka; 2 Department of Preventive and Restorative Dentistry, University of Sharjah, Sharjah, UAE; 3 Clinical Sciences Department, College of Medicine, University of Sharjah, Sharjah, UAE; 4 The University of Hong Kong, Hong Kong Special Administrative Region, China

**Keywords:** gut microbiota, spinal nerve injury, neuropathic pain, gut–brain axis, immunity

## Abstract

Environmental stressors can disrupt the gut–brain relationship and alter the gut microbial composition, potentially leading to chronic pain, including neuropathic pain (NP). To understand this complex relationship, we conducted a systematic scoping review to examine the gut microbial-neuroimmune connection to NP and the potential therapeutic targets. The review includes English-language manuscripts in databases such as MEDLINE, Cochrane, and DOAJ between January 2000 and April 2022. Out of the 48 full texts examined, only 15 articles met the inclusion criteria. These included a randomised controlled trial involving 327 individuals, an in vitro, and 13 animal model studies. The findings suggest that the gut flora plays a role in the immunological, neurological, and metabolic signalling pathways associated with NP. Animal studies have been the primary focus in this area, indicating that an imbalanced-gut microbiome and subsequent activation of biochemical and neuro-immunologic pathways may influence the development of NP. This review provides a comprehensive summary of the gut microbiome-immune-NP axis and identifies potential therapeutic targets. However, since most of the evidence comes from animal studies, future research should include clinical trials to gain a better understanding of the role of gut microbiota in NP and discover new therapeutic strategies.

## Introduction

The human gut is home to a rich microbial community of over 100 trillion microbes comprising bacteria, fungi, archaea, viruses, and protozoa that have numerous central effects on the host’s health (Feng et al., 2018). The gut microbiome has evolved alongside humans over the millennia, and it is now known that alterations in the gut environment caused by various stimuli such as diet and medications can beneficially or adversely affect the host’s health (Jalanka-Tuovinen et al., 2011). Energy regulation, gut barrier integrity, protection from pathogens, brain development, and immune system function all appear to depend on homeostasis between the gut microbiota and the host (Jalanka-Tuovinen et al., 2011; Belkaid and Hand, 2014). It is now known that dysbiosis of the gut microbiota and the host can lead to several diseases, including metabolic (Mazidi et al., 2016), cardiovascular (Peng et al., 2018), neurological abnormalities (Park and Kim, 2021), and gastrointestinal disorders (Ng et al., 2018).

Additionally, the microbiome–gut–brain axis appears to be a bidirectional signalling network connecting the brain and the gut microbiota via various neural, neurotransmitter, and molecular signalling mechanisms and pathways, as well as immunological axes (Cryan et al., 2019). Thus, the realisation of the significant role of gut microbiota in pain control, in particular, has recently aroused much excitement (Wang and Kasper, 2014). For instance, the gut microbiota appears to play a critical role in visceral or abdominal pain provocation. In addition, their role in inducing other types of chronic pain, including inflammatory and NP, and opioid tolerance, has been recognised (Amaral et al., 2008; Wang and Kasper, 2014).

A growing body of data indicates that gut microbiota could directly trigger nociceptors via their products and/or constitutive components. For instance, nociceptor neurons are susceptible to bacterial constitutive/secretory metabolites involved in pain signalling (Defaye et al., 2020). In this context, many contemporary studies have focused on neuropathic pain (NP), which is a direct outcome of damage or injury to the nervous system or dysfunction. The latter should be differentiated from nociceptive pain due to the damage to body tissues (Cohen and Mao, 2014).

Chiu et al. (2013) noted that bacterial formyl peptides cause mechanical pain sensitivity in mice by inducing calcium flow and action potentials in nociceptor neurons during infection. Additionally, they recently reported that pathogenic viral and fungal flora could modify nociceptor pain sensitivity through immune activation pathways (Chiu, 2018). Thus, gut microbiota appear to contribute to both NP as well as nociceptor pain.

In terms of the nature of the pain sensation, NP is often described as electric-like, stabbing, or lancinating. It is frequently accompanied by sensory deficiencies (numbness or tingling) and autonomic symptoms (Cohen and Mao, 2014). Physiological processes in NP include neuronal affections due to inflammation and spontaneous discharge of injured neurons (increase in Type III sodium channels and subunit α2δ calcium channel; decrease in opioid receptors) (Patel and Dickenson, 2016).

The foregoing implies that the gut microbiota is a critical regulator of neurological, immunological, and metabolic signalling pathways, contributing to a complex network that leads to the eventual development of NP (Lin et al., 2020). Hence in this scoping review, we investigated the current theories on gut microbial-neuroimmune connection related specifically to NP and attempted to identify some potential therapeutic targets for pain alleviation.

## Methods

### Data sources

Three reviewers (GN, KSF, and LPS) executed an electronic data search using MEDLINE, Cochrane, and DOAJ databases for the English language manuscripts. Published reports were accessed between 1 January 2000 and 30 April 2022 to identify studies evaluating the relationship between gut microbiota, neural-immune connection, and NP.

### Study selection

#### Inclusion criteria

- *Study design*: studies involving human participants or preclinical studies, including animal models lab experiments; molecular data; in vitro studies.
- *Population:* patients or animal models with NP.
- *Setting*: Clinics and lab settings.
- Date or country enforced no limitations.

#### Exclusion criteria

- Reports that present incomplete outcome details.
- Studies evaluating patients or animal models with other chronic pain conditions such as inflammatory pain, visceral pain, and headache.
- Studies that do not meet the set study objectives, grey literature, abstract only.

Following the PRISMA-ScR checklist containing 20 essential reporting items by Tricco et al. (2018) which included JBI-guided methodology, we attempted to create a comprehensive evidence synthesis about NP interlinked with the gut microbiome-neuroimmune system. Our inclusion criteria allowed for articles on Gut bacteria, the immune system, neural signal pathways, and Antiviral-medication related to NP enabling us to provide evidence mapping and conduct future-focused systematic reviews.

#### Search terms

A particular search string was set up for each of the databases, Table 1.

**Table 1.** Employed search terms in the Databases

Database	Search terms
Search in PubMed (MEDLINE) database	(“microbiota”[tiab] OR “bacteria”[tiab] OR “gut-microbiome” OR gut-bacteria OR (“neuropathic pain”[mesh])) (“chemo”[tiab] OR “chemo-drug”[tiab] OR “chemotherapy” OR “chemotherapy-induced” OR (“neuropathic pain”[mesh])) (“probiotic”[tiab] OR “prebiotic”[tiab] OR “synbiotic”[tiab] OR “bacteria-probiotic”[tiab] OR “postbiotic”[tiab] (“neuropathic pain”[mesh])) (“nerve constriction injury”[tiab] OR “chronic nerve injury”[tiab] OR “nerve injury”[tiab] OR “CCI”[tiab] “hyperalgesia”[tiab] OR (“neuropathic pain”[mesh])) (“spinal injury”[tiab] OR “spinal nerve”[tiab] OR “spinal nerve injury”[tiab] OR “SNI”[tiab] OR “hyperalgesia”[tiab] OR (“neuropathic pain”[mesh])) (“spared nerve”[tiab] OR “spared-nerve”[tiab] OR “spared nerve injury”[tiab]” OR (“neuropathic pain”[mesh])) (“neuropath”[tiab] OR “neuropathy”[tiab] “hyperalgesia”[tiab]) (neuralgia[mesh] OR “neuralgi”[tiab]) (“pain”[tiab] OR pain[mesh]) (((((“Human Studies”[mesh] OR “in vivo”[mesh]) OR “in vitro”[mesh] OR “animal model”[tiab]) OR “randomised controlled”[tiab]) OR RCT[mesh] case report[pt] OR case series[pt] review[pt] ((1 AND 2 AND 3 AND 4 AND 5 AND 6) OR (1 AND 3)) ((1 AND 2) OR (2 AND 3)) ((4 AND 5 AND 6) OR (7 AND 8 AND 9)) (4 AND 5 AND 6) OR (animals[mh] NOT humans[mh])) (7 AND 8 AND 9) (10 OR 11 OR 12) Limit: English
Cochrane Library database	(“neuropath”):ti,ab,kw AND (“microbiota”):ti,ab,kw (“neuropathic pain”):ti,ab,kw AND (“bacteria”):ti,ab,kw (“nerve pain”):ti,ab,kw AND (“gut microbe”):ti,ab,kw (“neuralgi”):ti,ab,kw AND (“gut bacteria”):ti,ab,kw (“neuropathy”):ti,ab,kw AND (“gut microbiome”):ti,ab,kw (“neuropath”):ti,ab,kw AND (“probiotics”):ti,ab,kw (“neuropathy”):ti,ab,kw AND (“prebiotics”):ti,ab,kw (“neuropathic”):ti,ab,kw AND (“synbiotic”):ti,ab,kw (“neuropathic pain”):ti,ab,kw AND (“postbiotic”):ti,ab,kw (“neuralgia”):ti,ab,kw AND (“chemotherapy”):ti,ab,kw (“neuropathy”):ti,ab,kw AND (“chemo-induced”):ti,ab,kw (“neuropathic pain”):ti,ab,kw AND (“chronic constriction injury”):ti,ab,kw (“neuropathy”):ti,ab,kw AND (“nerve-CCI”):ti,ab,kw (“neuropathic pain”):ti,ab,kw AND (“chronic constriction nerve injury”):ti,ab,kw (“neuralgia”):ti,ab,kw AND (“spinal injury”):ti,ab,kw (“hyperalgesia”):ti,ab,kw AND (“spinal nerve injury”):ti,ab,kw (“neuropathic pain”):ti,ab,kw AND (“SNI”):ti,ab,kw: (“hyperlagesia”):ti,ab,kw AND (“spared nerve injury”):ti,ab,kw: (((1 OR 3) AND 3 AND 5) OR (5 AND (1 OR 2))) (((1 OR 6) AND 7 AND 8) OR (5 AND (6 OR 7))) (((9 OR 11) AND 10 AND 12) OR (13 AND (11 OR 12))) (((15 OR 17) AND 116 AND 18) OR (14 AND (13 OR 15))) (((17 OR 18) AND 4 AND 5) OR (17 AND ( 1OR 5)))

#### Summary measure

The intended outcome was to examine the gut microbiota-immune-neural relationship in NP and prospective therapeutic targets for regulating variables.

### Electronic data search and analysis

**Figure 1.**
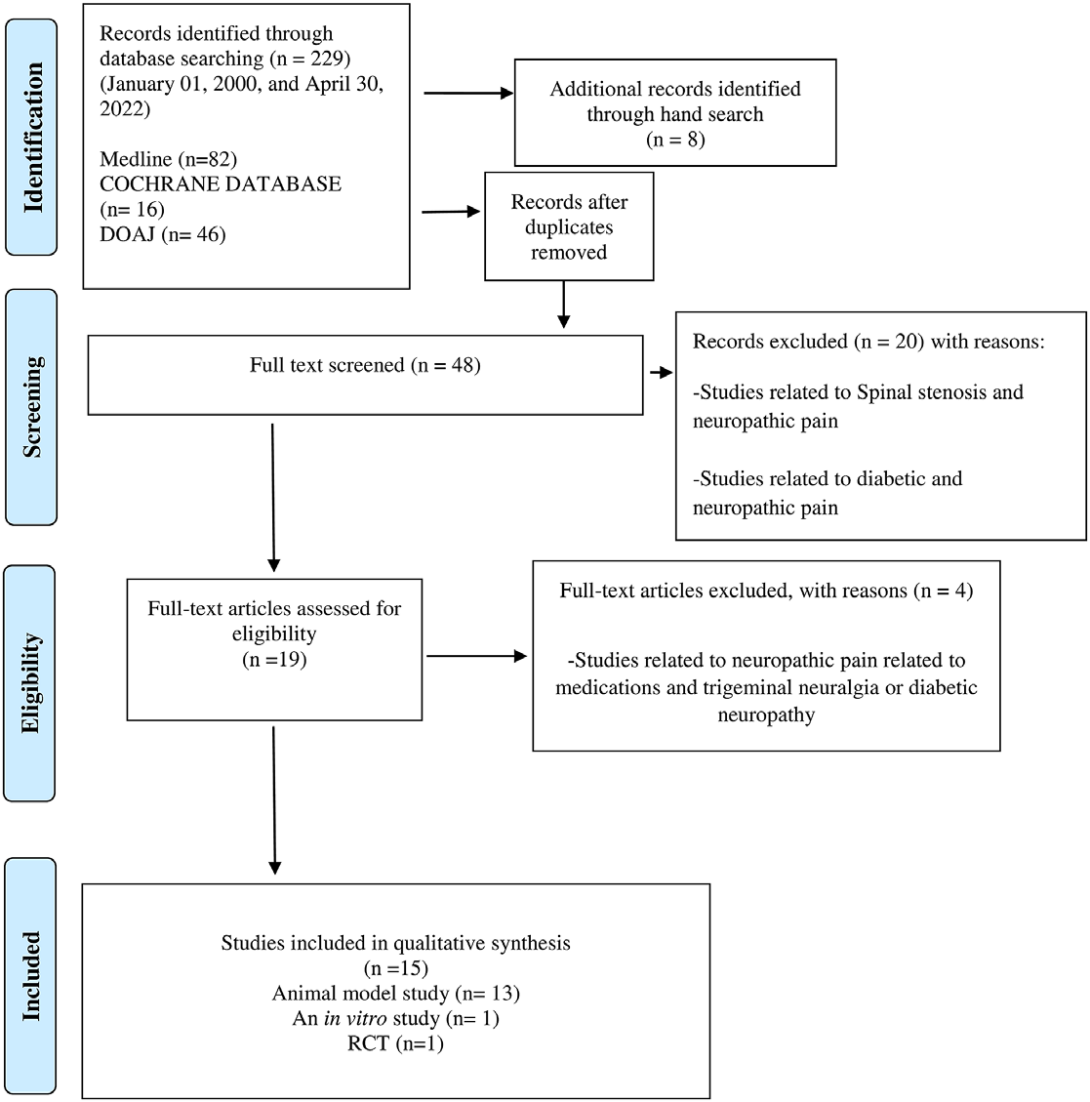
PRISMA flow chart of the literature search and study selection.

We designed and reported the present Scoping Reviews (ScR) in line with the Preferred Reporting Items for Systematic Reviews and Meta-Analyses (PRISMA) guidelines for a systematic and comprehensive approach (Tricco et al., 2018). Using a PRISMA flowchart (Figure 1), we describe the number of evidence sources screened, evaluated for eligibility, and included in the systematic scoping review, along with the reasons for exclusions. Two reviewers (GN and KSF) examined the titles and abstracts of all published relevant reports that met our predefined inclusion criteria during the first stage of the three-stage electronic data search and analysis. Afterwards, a full-text review of all relevant articles was conducted to thoroughly explore the data during stage two of the review process. A thorough examination of the full text of the selected literature confirmed that the eligibility criteria were met and that the reported outcomes correspond to the pre-set outcome measures. Additionally, we conducted a backward search of the references of the included reports. Finally, the reviewers (GN and KSF) extracted and assessed the data during stage two.

After the full-text review was completed, specific points related to the characteristics of each included study were mapped and recorded. This aided in classifying the study’s design, setting, intervention, and reporting jurisdiction. The sample size, assessment duration, evaluation methods, and study conclusions were analysed in detail. Finally, the third reviewer (LPS) verified the data accuracy by cross-checking these. We reviewed relevant studies on gut microbiota and NP. The identified manuscripts were catalogued using the bibliographic software tool Endnote version 20 (Clarivate Analytics, Chandler, AZ). The following table summarises the studies’ characteristics (Table 2).

**Table 2.** Major characteristics and results of the included studies

Study Country	Study type	Study intervention	Microbiota involvement
Nerve injury and neuropathic pain			
Chen et al. (2021) China	Pre-clinical study (animal model)	16S rDNA amplicon sequencing and serum and spinal cord metabolomics were used to identify microbiota and metabolite changes in sham (control) and CCI (Chronic Constriction injury) model rats	The gut microbiota composition differed between CCI-induced neuropathic pain rats and sham controls There was an increase in *Helicobacter, Phascolarctobacterium, Christensenella, Blautia, Streptococcus, Rothia*, and *Lactobacillus* In contrast, a decrease in the abundance of *Ignatzschineria, Butyricinomonas, Escherichia, AF12*, and *Corynebacterium* Additionally, 72 serum metabolites and 17 spinal cord metabolites differed significantly between CCI and sham rats
Brandon-Mong et al. (2020) Taiwan	Pre-clinical study (animal model)	Sixty-six faecal samples from three mice and at 11-time points in the spared nerve injury-induced neuropathic pain (SNI-NP) and Sham groups were analysed The 16S rRNA gene was PCR amplified and sequenced on a MiSeq platform	SNI-NP changes gut microbial diversity; the microbial community in the gut micro-ecosystem was affected by SNIP-NP In the Sham group, *Oscillospira*, which was classified as a low-abundance and core microbe, was identified as the primary microbe In contrast, in the SNI-NP group, *Staphylococcus* – classified as a rare and non-core microbe, was identified as the primary microbe
Yang et al. (2019) China	Pre-clinical study (animal model)	Comparing the gut microbiota makeup of the three groups (sham, anhedonia susceptible, and resilient rats) using 16S rRNA gene sequencing	The anhedonia-vulnerable gut microbiota differed from the sham-operated and resilient gut microbiota There was pain, depression, and anhedonia in antibiotic-treated rats, suggesting that the gut microbiota is responsible for these atypical behaviours Transplanting anhedonia-prone rat faeces into antibiotic-treated pseudo-germ-free mice increased pain and depression-like characteristics, including anhedonia However, transplanting resilient rat faeces into antibiotics-treated pseudo-germ-free mice improved pain and depression-like behaviours, including anhedonia
Drug-induced neuropathic pain			
Ellis et al. (2022) United States	Clinical study (Human)	Comparing gut microbial diversity and dysbiosis in people with HIV (PWH) and people without HIV (PWoH) with distal neuropathic pain (DNP). The gut microbiome was characterised using 16S rRNA sequencing, and diversity was assessed using phylogenetic tree construction	In contrast to PWoH in PWH, more severe DNP was related to reduced alpha diversity measured by Faith’s phylogenetic diversity Gut dysbiosis, specifically reductions in diversity and relative increases in Blautia and Clostridium to Lachnospira ratios plays a role in the prevalence of DNP in PWH
Ramakrishna et al. (2019) United States	Pre-clinical study (animal model)	129SvEv and C57BL/6 mice are sensitive and resistant to Paclitaxel-induced discomfort, respectively Reciprocal gut microbiota transfers and antibiotic depletion in B6 and 129 mice with changes in Paclitaxel-induced pain sensitivity	Microglia increased in the spinal cords of Paclitaxel-treated mice with the pain-sensitive B6 microbiota but not the pain-resistant 129 microbiota, which had no immune cells invading Microglia are causally engaged in Chemotherapy-induced peripheral neuropathy (CIPN), and gut bacteria are the drivers of this phenotype
Shen et al. (2017) United States	Pre-clinical study (animal model)	Mechanical hyperalgesia in germ-free (GF) and specific pathogen-free (SPF) mice treated with oxaliplatin	Oxaliplatin-induced neuropathic pain was reduced in GF mice and antibiotic-treated mice Restoring the microbiota of GF mice abolished the effects
Gut-neuroimmune crosstalk in neuropathic pain			
Wardill et al. (2016) Australia	Pre-clinical study (animal model)	The gut microbiome profile of control animals was determined through bacterial faecal sample sequencing TLR4 deletion reduces chemotherapy-induced gastrointestinal damage, and the pain was assessed in an animal model	Chemo-drug-Irinotecan-induced gastrointestinal toxicity and pain were reduced in TLR4-deficient mice
Costigan et al. (2009) United Kingdom	Pre-clinical study (animal model)	In response to the sparing nerve injury (SNI) model of peripheral neuropathic pain, compared the gene expression profiles in the spinal dorsal horn of adult and newborn rats	Compared to neonatal, in adult rats, spinal cords indicate a greater microglial and T-cell response Following peripheral nerve injury in the dorsal horn of the spinal cord, T-cell infiltration and activation promote the evolution of neuropathic pain-like hypersensitivity in adult rats than neonatal rats
Potential therapeutic targets of neuropathic pain			
Cuozzo et al. (2021) Italy	Pre-clinical study (animal model)	Each of the four experimental groups utilised ten animals (40 animals) in total: *G1*: Vehicle-treated Sham Mice (*n* = 10) *G2*: Probiotic-treated sham mice (*n* = 10) *G3*: Paclitaxel + Vehicle (*n* = 10) *G4*: Paclitaxel + probiotics (*n* = 10)	In an ex vivo analysis, probiotic administration boosted the expression of opioid and cannabinoid receptors in the spinal cord, avoided nerve fibre injury in the paws, and altered serum pro-inflammatory cytokines concentration in CIPN-mice Probiotic administration maintained gut integrity and function. Also, probiotics reduced mechanical and cold hypersensitivity in paclitaxel mice
Lian et al. (2021) China	Pre-clinical study (animal model)	All C57BL/6J mice were sorted into four groups: *G1*: Regular water and Saline (H_2_O + Saline) *G2*: Regular water and oxaliplatin (H_2_O + OXA) *G3*: Hydrogen-rich water and saline injections (HW + Saline) *G4*: Hydrogen-rich water plus oxaliplatin (HW + OXA)	Hydrogen-rich water may diminish oxaliplatin-induced hyperalgesia, modify gut microbiota diversity and structure, reverse inflammatory cytokines and oxidative stress, and lower LPS and TLR4 expression
Ding et al. (2021) United States	Pre-clinical study (animal model)	Broad-spectrum antibiotics/water were given to perturbed gut microbiota in mice, CCI in the intervention group, and water in the control mice The spinal cord infiltrating T cells was characterised by examining interferon (IFN)-γ, interleukin (IL)-17, and Foxp3, depleting regulatory T cells	Oral antibiotics altered gut microbiota and slowed CCI neuropathic pain development Changes in gut microbiota skew immune profiles from pro-inflammatory to anti-inflammatory Foxp3+ regulatory T cells were protective against neuropathic pain mediated by gut microbiota changes, but their depletion increased IFN-producing Th1 cell infiltration in the spinal cord
Castelli et al. (2018) Italy	In vitro study	Multistrain Probiotic Product (De Simone Formulation)-DSF probiotic formulation in an in vitro model of a sensitive neuron, the F11 cells	A probiotic formulation reduced Paclitaxel-induced neuropathic pain
Austin et al. (2012) Australia	Pre-clinical study (animal model)	In two neuropathy models, sciatic nerve chronic constriction injury and experimental autoimmune neuritis (EAN) in rats, the effects of increasing regulatory T cells (Tregs) on pain hypersensitivity and neuroinflammation were investigated	Tregs play a key role in endogenous recovery from neuropathy-induced pain In nerve-injured and EAN-affected rats, boosting nTregs with CD28SupA lowers neuroinflammation and pain hypersensitivity, but lowering nTregs with an anti-CD25- depleting antibody enhances pain hypersensitivity in nerve-injured mice
Bráz et al. (2012) United States	Pre-clinical study (animal model)	Immature telencephalic GABAergic interneurons from the mouse medial ganglionic eminence (MGE) were transplanted into the adult mouse spinal cord	The MGE-derived GABAergic interneurons can reverse the mechanical hypersensitivity caused by peripheral nerve injury. Thus, they can be used to alleviate spinal cord hyperexcitability in neuropathic pain
Sommer et al. (2001) Germany	Pre-clinical study (animal model)	Mice were given etanercept or sham treatment by local nerve injection to the injured nerve or by systemic application to study whether the etanercept can reduce neuropathic pain in chronic constriction injury of sciatic nerve animals model	Etanercept competitively inhibits tumour necrosis factor-alpha (TNF) Etanercept significantly reduced mechanical allodynia and thermal hyperalgesia in both application methods, suggesting a therapeutic option for neuropathic pain

### Quality and the overall risk of bias assessment of the included reports

During the third stage of the systematic review of available records, two reviewers (LPS and KSF) independently assessed the quality of eligible studies using the SYRCLE’s risk of bias tool for animal studies, which has ten components (Hooijmans et al., 2014). These components pertain to six different types of bias: selection bias, performance bias, detection bias, attrition bias, and reporting bias.

To assign a judgement of low, moderate, or unclear risk of bias to each of the 10 tool items, we followed the provided detailed list with signalling questions. A “yes” judgement indicates a low risk of bias; a “no” assessment suggests a high risk of bias, and the decision will be “unclear” if insufficient details are provided. Two independent reviewers conducted the assessments (GN and KSF), and all involved authors (GN, KSF, LPS, HCN) cross-checked the scores. Any disagreements were resolved through consensus-building discussions with the third and fourth reviewers (LPS and HCN). All reviewers involved discussed their decisions based on the cumulative scores. Low-risk reports contain a higher percentage of “yes” scores, moderate-risk reports include an “unclear” score, and high-risk reports have a higher percentage of “no” scores. So, we adopted an ≥80% score as low-risk and the risk score of ≤50% was considered high-risk as the cut-off point to include or exclude the study (Table 3).

**Table 3.** Risk of bias appraisal of the reviewed studies

Studies	Selection bias			Performance bias		Detection bias		Attrition bias	Reporting bias	Other
	Sequence generation	Baseline characteristics	Allocation concealment	Random housing	Blinding	Random outcome assessment	Blinding	Incomplete outcome data	Selective outcome reporting	Other sources of bias
Chen et al. (2021)	UC	Y	Y	Y	UC	Y	UC	Y	Y	Y
Ding et al. (2021)	UC	Y	Y	Y	UC	Y	UC	Y	Y	Y
Lian et al. (2021)	Y	Y	Y	Y	Y	Y	UC	Y	Y	Y
Cuozzo et al. (2021)	Y	Y	Y	Y	UC	Y	UC	Y	Y	Y
Brandon-Mong et al. (2020)	Y	Y	Y	Y	UC	Y	UC	Y	Y	Y
Yang et al. (2019)	Y	Y	Y	Y	Y	Y	UC	UC	UC	Y
Ramakrishna et al. (2019)	N	Y	Y	Y	Y	Y	UC	Y	Y	Y
Shen et al. (2017)	Y	Y	Y	Y	Y	Y	UC	Y	Y	Y
Wardill et al. (2016)	UC	Y	Y	Y	Y	Y	UC	Y	Y	Y
Castelli et al. (2018)	UC	Y	Y	Y	UC	Y	UC	Y	Y	Y
Austin et al. (2012)	Y	Y	Y	Y	Y	Y	UC	Y	Y	Y
Bráz et al. (2012)	Y	Y	Y	Y	UC	Y	UC	Y	Y	Y
Costigan et al. (2009)	UC	Y	Y	Y	Y	Y	UC	Y	Y	Y
Sommer et al. (2001)	Y	Y	Y	Y	Y	Y	UC	Y	Y	Y

N, no (high risk of bias); UC, unclear (moderate risk of bias); Y, yes (low risk of bias).

## Results

Of the 48 full texts reviewed, only 15 articles met the set inclusion criteria (Figure 1). The reviewed articles encompassed 13 studies using various animal models (Sommer et al., 2001; Gwak et al., 2006; Costigan et al., 2009; Austin et al., 2012; Bráz et al., 2012; Wardill et al., 2016; Shen et al., 2017; Ramakrishna et al., 2019; Yang et al., 2019; Brandon-Mong et al., 2020; Chen et al., 2021; Cuozzo et al., 2021; Ding et al., 2021; Lian et al., 2021), a single in vitro study (Castelli et al., 2018), and a single randomised controlled trial (RCT) with 327 enrolled participants, Table 2 (Ellis et al., 2022).

### Gut microbes and CNS link

Over the last few years, a number of studies have revealed that gut microbiota potentially plays a role in pain and `depression-like` phenotypes. For instance, Yang et al. (2019) observed susceptibility to anhedonia (inability to feel pleasure) following partial or spared nerve injury (SNI) in a rat model, which they surmised was linked to dysbiotic attributes of the gut microbiota. Furthermore, faecal microbiota transplantation from anhedonia-susceptible rats into antibiotics-treated, pseudo-germ-free mice significantly increased pain and depression-like behaviour, including anhedonia. Conversely, transplanting faecal microbiota from healthy rats into antibiotics-treated pseudo-germ-free mice substantially alleviated pain and depression-like behaviour, including anhedonia. These observations indicate that the aberrant composition of gut microbiota may play a modulatory role in pain and depression-like phenotypes, including anhedonia, thus showing the importance of the gut microbial and brain axis (Yang et al., 2019).

#### Nerve injury-induced altered gut microbiota and their metabolites in modulating NP

Brandon-Mong et al. (2020) were the first to suggest that spinal nerve injury (SNI) may modulate the stability of the core microbiota of the gut. On comparing the gut ecosystem of an SNI and a sham group of mice, they noted that the sham group had a stable microbiome with a predominance of the key genus of bacteria belonging to the genus *Oscillospira*, known to produce short-chain fatty acids (SCFA) – butyrate, and anti-inflammatory and neuroprotective – metabolites. While in contrast, the genus *Staphylococcus*, known for its notorious inflammatory effects, was identified as the predominant microbe in the SNI group (Brandon-Mong et al., 2020).

Similarly, Chen et al. (2021), in a subsequent study of experimental mice with chronic constriction injury (CCI)-induced NP and sham controls, also demonstrated altered gut microbiota between the two groups, with the beneficial, *Butyricimonas* genus, which produces an SCFA butyrate being diminished in the former group. They further noted an abundance of the *Helicobacter* genus in the CCI group, which correlated with increased levels of serum l-tyrosine and dopamine, which are important chemical mediators associated with pain transmission (Chen et al., 2021).

#### Drug-induced gut dysbiosis and metabolites in modulating NP

Mounting evidence indicates that drug-induced shifts of the gut microbiota may trigger the development of NP. Essentially this entails qualitative and quantitative changes in the gut microbiota, whence a commensal, symbiotic state turns into a dysbiotic state due to the impact of either antibiotic, antiviral, or chemotherapeutic drug.

*Antibiotics:* The first report of the link between antibiotics and NP was from Shen et al. (2017), who noted the alleviation of chemotherapy-induced mechanical hyperalgesia in a mouse model pretreated with antibiotics. They observed that the mechanical hyperalgesia produced by chemotherapeutic – Oxaliplatin was ameliorated in germ-free (GF) mice and/or those pretreated with antibiotics. Furthermore, they discovered that the aggregation of macrophages and cytokines in the dorsal root ganglia (DRG) was significantly reduced in antibiotic-treated mice, demonstrating connectivity between the inflammatory response and the gut microbiota. Moreover, a return to a eubiotic and healthy composition of the gut microbiota was associated with a concomitant attenuation of the inflammatory response, as well as NP (Shen et al., 2017).

*Antivirals:* In a very recent study, Ellis et al. (2022) demonstrated dysbiosis of the gut microbiota in a Human Immunodeficiency Virus (HIV) – infected cohort on antiretroviral therapy in comparison to a matched cohort of healthy individuals. They noted that higher NP was associated with decreased microbial diversity and a relatively high predominance in the ratios of *Blautia* and *Clostridium* species to *Lachnospira* species in the HIV-infected individuals. The relative reduction of *Lachnospira* in the latter group appeared to have aggravated nerve damage and contributory inflammation. Interestingly, the latter species are known to produce neuro-beneficial metabolites such as small chain fatty acids (SCFA), mentioned above. Thus, they concluded that the composition and diversity of gut microbes might modulate NP and potentially contribute to pain sensation in general (Ellis et al., 2022).

*Chemotherapeutics:* Paclitaxel, a class of chemotherapeutic medications called anti-microtubule agents, works by stopping the growth and spread of cancer cells. Ramakrishna et al. (2019) noted in a mouse model that Paclitaxel reduces the number of beneficial bacteria in drug-treated mice’s gastrointestinal (GI) tract. For instance, *Akkermansia muciniphila*, which is known to improve the barrier function of the intestine, was notably reduced in the latter group. In addition, this commensal promotes mucus production, intestinal epithelial-cell renewal, and beneficial metabolites, such as short-chain fatty acids (SCFAs), all of which contribute to a healthy gut ecosystem (Ramakrishna et al., 2019).

#### Gut-neuroimmune crosstalk and immune targets in controlling NP

The central nervous system (CNS) is now recognised as critical in modulating both central and peripheral immune responses. In particular glial cells of CNS, especially microglia, appear to react to innate immune responses. This mechanism called the neuroimmune crosstalk, is maintained during neurodevelopment but is still largely unexplored. Below, we discuss the relationship between the gut microbiota and the NP as well as the effectiveness of pharmaceuticals in regulating NP via immunological targets.

*Chemokine–cytokine cascade – TNFα and NP:* Peripheral nerve damage initiates a cascade of immunological reactions. As a result, there is macrophage infiltration, activation of T cells, and increased expression of proinflammatory cytokines such as IL-1 and IL-6, and TNFα (Sudo et al., 2004; Fregnan et al., 2012) Etanercept, a competitive inhibitor of TNFα, was tested by Sommer et al. (2001) for its ability to reduce pain and hyperalgesia. They induced neuropathy by chronic constriction injury in the sciatic nerve of mice and noted that preventive treatment with the TNFα-sequestering agent Etanercept reduces hyperalgesia (Sommer et al., 2001)

In this context, Cuozzo et al. (2021) recently employed a mouse model to compare the benefits of a probiotic formulation of *bifidobacteria* and *lactobacilli* and the aforementioned chemotherapeutic Paclitaxel. Compared to the chemotherapeutic drugs, probiotics reduced colon tissue damage, increased cannabinoid, and opioid expression in the spinal cord, decreased inducible nitric oxide synthase, and decreased TNF- α, IL-1, and IL-6 serum levels (Cuozzo et al., 2021).

*T cells (Tregs) and NP:* In their SNI experimental model, Costigan et al. (2009) found that mice lacking mature T-cells had a lesser degree of hyperalgesia. Furthermore, immunohistochemical studies of the spinal dorsal horn showed T-cell infiltration and positive staining for Iba-1 and CD2 in test mice. This suggests that immunological pathways may have a dampening effect on NP (Costigan et al., 2009). Another animal model NP by Ding et al. (2021) showed that gut microbiota influences T-cell immune responses induced by CCI. Thus, demonstrating that the latter is inextricably linked to the induction of NP through the T-cell response pathway.

The link between the gut microbiota, NP, and immune pathways is further illustrated in the above study by Ding et al. (2021) where administration of oral antibiotics led to a dysbiotic gut microbiota which in turn dampened mechanical allodynia and thermal hyperalgesia, caused by nerve injury. However, these symptoms were alleviated after the reversal of dysbiosis to eubiosis. The researchers, therefore, postulated that the gut microbiota exerted a protective role mediated by anti-inflammatory regulatory T cells (Tregs).

In another interesting study, Austin et al. (2012) used CD28 super-agonist (CD28SupA), a T-regulatory cells (Tregs) population expander, as a therapeutic method to control NP in another animal model of CCI injury. CD28SupA significantly increased Tregs in the lymphoid tissues in the control group, which correlated with reduced numbers of T cells, macrophages, and antigen-presenting cells in the dorsal root ganglia and the sciatic nerve. Also, there were reduced numbers of microglial cells and infiltration of T cells in the spinal cord. Furthermore, depletion of Tregs by a CD25 antibody in mice with a partial sciatic nerve ligation resulted in prolonged mechanical pain hypersensitivity. These findings suggest that Tregs play a role in endogenous recovery from neuropathy-induced pain, implying that the T-cell subset may be specifically targeted to alleviate chronic NP (Austin et al., 2012)

*Gut microbial lipopolysaccharide (LPS), immune cell Toll-like receptors (TLRs), and NP:* Lipopolysaccharides (LPS, also termed endotoxins) are intrinsic cell wall constituents of Gram-negative bacteria. On the other hand, TLRs are a family of transmembrane protein receptors that recognise microbes by binding to pathogen-associated molecular patterns (PAMP). For example, TLR4 can recognise the endotoxins of gut bacteria and, when activated, initiate a downstream signalling pathway leading to the secretion of proinflammatory cytokines and chemokines (Wardill et al., 2015). The work by the latter group on chemotherapy-induced NP (CINP) in mice model (2016) has elegantly demonstrated that TLR4 induces an exacerbated innate immune response resulting in an altered toxicity profile in these animals. Furthermore, genetic deletion of TLR4 from mice improved chemo drug-induced gut toxicity as well as NP (Wardill et al., 2016). Thus, an intact or eubiotic gut microbiome helps immune homeostasis, alleviating NP through a microbiota-mediated signalling pathway/s.

A recent experiment by Lian et al. (2021) in a mouse model also indicates that Hydrogen-rich water therapy might suppress the LPS-TLR4 pathway by controlling the number and diversity of gut microbiota. This process appears to improve chemotherapeutic agent, Oxaliplatin-induced mechanical hyperalgesia, and reverse the imbalance of inflammatory cytokines and oxidative stress, thus alleviating CINP (Lian et al., 2021).

Akin to enterocyte-expressed TLR4 in gut inflammation, glial cells expressed TLR4 also in CNS, thus modulating NP. Ramakrishna et al. (2019) provide compelling evidence that spinal microgliosis is causally implicated in the chemotherapeutic agent Paclitaxel-induced NP.

*Transient receptor potential (TRP) channels in Dorsal root ganglion and TLR4 in NP:* There are a number of other agents that leads to TRP channel activation, and these include bacterial LPS, polyunsaturated fatty acids, and various probiotic combinations. Thus, LPS induces nociceptive neuron depolarization via the TRP channel (which TLR4 also mediates), resulting in intracellular calcium accumulation and activation of nociception neurons (Boonen et al., 2018). Gut microbial metabolites such as polyunsaturated fatty acids (PUFAs) also elicit peripheral nerve hypersensitivity following TRP channel-TRPV4 activation (Michalak et al., 2016).

Castelli et al. (2018) reported in an in vitro study that administering a high-concentration probiotic containing 450 billion bacteria per sachet could mitigate chemotherapy-induced NP by lowering inflammatory signals and neutralising chemotherapeutic Paclitaxel-induced elevated TRPV1 and TRPV4 channels. Therefore, the reported DSF formulation is seemed an effective adjuvant therapy for reducing cold hypersensitivity caused by physical nerve injury or chemotherapy (Castelli et al., 2018).

*Gut bacteria and excitatory and inhibitory neurotransmitters in NP:* Gut bacteria can convert glutamate, an excitatory neurotransmitter, into gamma-aminobutyric acid (GABA), an inhibitory neurotransmitter. The transplantation of GABAergic precursor cells has been shown to correct allodynia in a mouse NP model, according to Bráz et al. (2012). Based on their observation, these researchers suggest that GABAergic precursor cell transplantation could be an alternate treatment in NP-related situations.

Additionally, neurons and glial cells regulate GABA levels (Hoshino, 2012). Gwak et al. (2006) used the spinal cord injury (SCI) model to explore central NP following SCI. They found that SCI caused hypofunction of GABAergic tone and caused glial activation. Therefore, they suggest that specific pharmacological or molecular suppression of glial activation can improve GABAergic tone and is a possible method for treating chronic NP (Gwak et al., 2006).

## Discussion

It has been known for over a decade that the metabolic activity of the gut microbiota could impact the functionality of distant organs, such as the brain, through critical communication axes (Carabotti et al., 2015). For example, in an exciting, mice-model experiment, Yang et al. (2019) demonstrated a bidirectional interaction in the microbiota–gut–brain axis where faecal microbiota transplantation from robust animals without anhedonia dramatically reduced the pain, depression, and anhedonia-like behaviours in mice with SNI.

**Figure 2.**
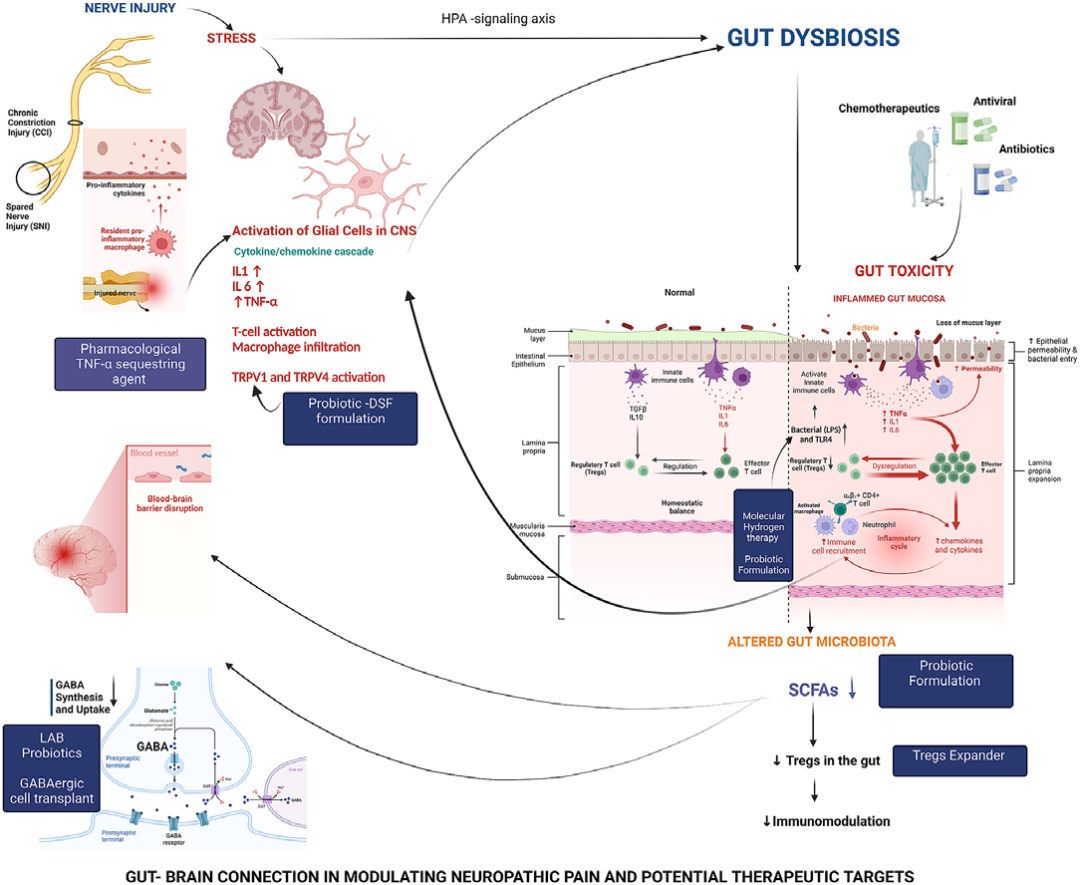
schematic diagram depicting the postulated pathways of gut-brain connectivity leading to neuropathic pain.

Although the exact processes and mechanisms underlying such gut microbiota and the brain crosstalk are still under investigation, signs are emerging of a number of pathways through which the gut microbiota can impact at least in some variants of pain sensations, such as NP (Figure 2). For instance, gut microbes can influence NP by modulating the immune system through synthesising several neurotransmitters, such as short-chain fatty acids (SCFAs), that have neuroprotective properties.

This review, for the first time, collates the significant findings appertaining to the gut microbiome -NP axis, and potential therapeutics in this context, as follows:

- Stress and nerve injury induced gut-dysbiosis and NP.
- Drug-induced gut dysbiosis NP.
- Gut bacteria and their metabolites in NP.
- Gut bacteria – excitatory and inhibitory neurotransmitters in NP.
- Gut-neuroimmune crosstalk and immune targets modulating NP.

### Stress and nerve injury induced gut-dysbiosis and NP

It is well documented that the CNS modulates emotions and stressful situations altering the gut physiology, including motility, secretions, blood flow, nociception, and immune function. Similarly, nerve injury could impact gut microbial composition via alterations in gut functionality, such as the release of neurotransmitters, motility, and intestinal immune tone (Rhee et al., 2009). For example, some two decades ago, Sudo et al. demonstrated that germ-free mice had an exaggerated hypothalamic-pituitary-adrenal (HPA) axis response to stress, which was reversed by gut colonisation with a specific bacterial species belonging to genus *Bifidobacteriaceae* (Sudo et al., 2004).

Apart from stress, nerve injury, and NP may induce microbial dysbiosis in the gut. The experimental animal models of Brandon-Mong et al. (2020) and Chen et al. (2021), where SNIP and CCI induced NP, respectively, clearly demonstrated qualitative shifts in the gut microbiota. For example, these workers noted a significant increase in the *Staphylococcus* species that are highly inflammogenic in their SNIIP animal models (Brandon-Mong et al., 2020; Chen et al., 2021). Blake et al. (2018) have commented that α-hemolysin, pore-forming toxins secreted by *Staphylococcus aureus* that induces neuronal firing and spontaneous pain, may have contributed to the NP in the experimental animals.

Other workers have also found that stressful situations can lead to alterations in the intestinal microbial community profile, including the main microbial phyla (Galley et al., 2014). A cascading knock-on effect of stress and NP is the impact on the functionality of the intestinal cellular components, such as epithelial cells, immune cells, smooth muscle cells, enteric neurons, enterochromaffin cells, and interstitial cells, have been described by Mayer et al. (2014).

In addition, neuro-pathophysiological conditions also activate the neuroendocrine stress axis with the activation of sympathetic and parasympathetic systems leading to the release of neurotransmitters that influence the mucosal barrier and indirectly undermine mucosal integrity (Li et al., 2020). A good example is cortisol, a primary stress hormone that weakens the mucosal barriers, leading to increased intestinal permeability (Carabotti et al., 2015). A secondary effect of this phenomenon is the translocation of intestinal microbiota and their metabolites across the `leaky` mucosal lining, into the systemic circulation, with concomitant localised or systemic inflammation (Gareau et al., 2008; Li et al., 2020)

### Drug-induced gut dysbiosis NP

Mounting evidence implies that gut dysbiosis due to various pharmacologic agents plays a critical regulatory role in the production of NP, either directly or indirectly (Lin et al., 2020). For instance, chronic pain is prevalent in human immunodeficiency virus (HIV)-infected individuals. One study has shown that antiretrovirals such as zidovudine and efavirenz against HIV have a secondary antibacterial effect against *Bacteroides fragilis* and *Prevotella,* which are major commensal constituents of gut microbiota in health. In addition, *Enterococcus faecalis,* another common commensal, was inhibited by the antiviral -Efavirenz (Ray et al., 2021). Hence some have suggested that neuroinflammation and chronic NP in HIV patients could be due to gut dysbiosis precipitated by antiretroviral therapy. In a recent study, Ellis et al. (2022) noted that a higher degree of NP in HIV subjects was associated with decreased microbiome diversity and a relative decrease in a specific bacterial species termed *Lachnospira.* A reason for this appears to be a relative reduction in the metabolites of *Lachnospira,* including short-chain fatty acids (SCFA), that have anti-inflammatory and neuroprotective effects (Markowiak-Kopeć and Śliżewska, 2020; Ellis et al., 2022).

Similar to antivirals, antibiotics can precipitate a dysbiotic microbiome leading to a dysfunctional gut microbial community. Shen et al. (2017) clearly demonstrated this phenomenon in a mice model; they noted that gut microbiota promotes the development of chemotherapy-induced mechanical hyperalgesia. Oxaliplatin-induced mechanical hyperalgesia was reduced in germ-free mice and mice pretreated with antibiotics. However, restoring the microbiota of germ-free mice abrogated this protection. They suggested that such effects may be mediated, in part, by TLR4 expressed on haematopoietic cells, including macrophages (Shen et al., 2017).

Chemotherapy-induced gut toxicity (CIGT) is also recognised as a factor contributing to severe gastrointestinal side effects. CIGT is characterised by severe ulceration, inflammation, pain, glial cell activation, and elevated proinflammatory cytokines (Lin et al., 2020). Ramakrishna et al. (2019) noted in a mouse model that the chemotherapeutic drug Paclitaxel reduces the number of beneficial commensal bacteria, such as *Akkermansia muciniphila*, known to facilitate gut barrier function. This organism promotes mucus production, intestinal epithelial-cell renewal, and many metabolites, such as SCFA, needed to maintain a healthy gut biome (Markowiak-Kopeć and Śliżewska, 2020). Thus, chemotherapy triggers barrier dysfunction, resulting in greater systemic exposure to toxic bacterial metabolites, enhancing systemic inflammation and pain sensitivity.

### Gut bacteria and their metabolites in NP

The commensal organisms in the gastrointestinal (GI) tract produce numerous metabolites via anaerobic fermentation of indigestible complex polysaccharides such as dietary fibre and resistant starch (Havenaar, 2011). As mentioned above, short-chain fatty acids (SCFAs), including acetate, propionate, and butyrate, are particularly noteworthy in this context as vital mediators of the gut–brain communication system (Havenaar, 2011). The altered SCFA production attests this in various neuropathological conditions (Ma et al., 2019; Metzdorf and Tönges, 2021; Mirzaei et al., 2021), leading to NP, as described by many (Brandon-Mong et al., 2020; Chen et al., 2021; Ellis et al., 2022).

So how does SCFA disrupt gut functionality and impact NP? First, butyrate modulates the immune function by playing a role in the differentiation of anti-inflammatory T regulatory cells (Treg) that controls interleukin secretion and modify NP (Bhaskaran et al., 2018). Second, SCFAs are known to improve gut health through focal effects ranging from maintaining intestinal barrier integrity and mucus production. Third, SCFA receptors are expressed in different cell types, including those of the gastrointestinal mucosa and the immune and nervous systems, the latter playing an essential role in moderating NP (Lin et al., 2020). Finally, SCFA appears to serve a vital function in preserving CNS homeostasis by maintaining the integrity of the blood–brain barrier (Dalile et al., 2019). For example, in one study, the permeability of cerebrovascular endothelial cells to the toxic lipopolysaccharides of the Gram-negative bacteria was mitigated by the SCFA propionate (Hoyles et al., 2018).

Several studies have also demonstrated that sodium butyrate inhibits microglial activation and proinflammatory cytokine production, thereby inducing NP (Huuskonen et al., 2004; Zhou et al., 2021). Similarly, treating primary microglia cultures in vitro with acetate lowers the expression of proinflammatory cytokines IL-1, IL-6, and TNF-α expression. This implies that the reduction of SCFA-producing microorganisms may lead to a concomitant reduction of NP by influencing microglial activation and the subsequent polarisation of proinflammatory cytokines (Lin et al., 2020).

To conclude, SCFA production in the gut depends on the quality and quantity of the gut microbiota, other environmental factors, and host nutrition (Markowiak-Kopeć and Śliżewska, 2020). Hence researchers who have evaluated the impact of gut microbiome-produced SCFA have suggested that probiotic therapy may be one way that can modulate NP (Markowiak-Kopeć and Śliżewska, 2020; Zhou et al., 2021).

### Gut bacteria: excitatory and inhibitory neurotransmitters in NP

Pain perception is associated with various neurotransmitters, which can be broadly divided into inflammatory and noninflammatory mediators (Gwak and Luo, 2022). Spinal cord injury (SCI) disrupts inhibitory output, sensitising spinal dorsal horn excitatory neurons and increasing nociceptive transmission. GABA is a key inhibitory neurotransmitter hypothesised to be important in spinal inhibitory synaptic transmission. Therefore, disruptions in GABAergic inhibitory output cause neuronal hyperexcitability in the spinal dorsal horn and chronic NP after SCI (Gwak et al., 2006; Gwak and Luo, 2022).

Several reports document that the most specific neurotransmitters in NP are glutamate and GABA, being neuroexcitatory and inhibitory, respectively (Gwak and Luo, 2022). GABAergic precursor cell transplantation fixes allodynia in a mouse NP model (Bráz et al., 2012).

Gut bacteria such as *Escherichia coli* and *lactobacilli* synthesise GABA (Cui et al., 2020). Hence, microbial engineering that promotes GABA production may serve as a potential next-generation probiotic technology in the context of NP regulation.

### Gut-neuroimmune crosstalk and immune targets for controlling NP

#### Chemokine–cytokine cascade and probiotics in NP

In NP studies, chemotherapy-induced gut toxicity (CIGT) led to an elevated level of proinflammatory cytokines (IL-1β IL-6, TNF-α). Also, CIGT disrupts intestinal epithelial integrity, resulting in the translocation of the gut microbiota (Logan et al., 2008). Furthermore, the gut microbial metabolites stimulate host antigen-presenting cells, provoking the generation of proinflammatory mediators that are now known to be essential mediators of NP (Logan et al., 2008).

Several studies indicate that inhibiting overexpressed chemokines and their receptors significantly alleviates NP (Sommer et al., 2001; Miller et al., 2009). For instance, probiotic formulations can regulate the immune system by increasing anti-inflammatory cytokines (IL-10, TGF-β) and decreasing the proinflammatory cytokines (including interleukins, TNF-α IFN-γ). For example, Cuozzo et al. (2021) reported that, in experimental mice, the administration of a probiotic formulation of *bifidobacteria* and *lactobacilli* led to high serum levels of proinflammatory cytokines TNF-α, IL-1, and IL-6. They also noted that probiotics reduced colon tissue damage, upregulated cannabinoid and opioid expression in the spinal cord, and decreased inducible nitric oxide synthase (iNOS), all of which help reduce NP (Cuozzo et al., 2021).

#### Chemokine–cytokine cascade: TNFα receptor inhibitors and NP

As noted, peripheral nerve damage initiates a cascade of immunological reactions (Miller et al., 2009), resulting in infiltration of macrophages, activation of T cells, and increased expression of proinflammatory cytokines, including TNFα (Totsch and Sorge, 2017). The latter has received considerable attention as a mediator of NP (Miller et al., 2009). Etanercept, a competitive inhibitor of TNFα has been evaluated for its ability to reduce pain and hyperalgesia in an animal model of painful neuropathy due to CCI of the sciatic nerve. Etanercept reduces hyperalgesia, though it has little impact once it develops (Sommer et al., 2001).

#### Microbiota-mediated immune signalling: LPS-TLR4 pathway

Disruption of the homeostasis of the gut microbiota as well as the innate host mucosal immune system has been shown to activate TLRs, a family of transmembrane protein receptors expressed in numerous cell types, including immune cells. TLRs recognise a diverse range of danger signals from noxious substances and activate the protective innate immune system. Of the many TLR subtypes, TLR4 is the most extensively characterised and has a well-established role in the host immune response (Wardill et al., 2015). For example, TLR4 can recognise the endotoxins and recruit adaptor molecules and kinases, thus, initiating a downstream signalling cascade that culminates in the secretion of proinflammatory cytokines and chemokines (Wardill et al., 2015). Wardill et al. (2016) have demonstrated that TLR4- induces an exacerbated innate immune response resulting in a high toxicity profile in a CINP mice model, while deletion of the *TLR4* gene in mice improved chemotherapy-induced gut toxicity as well as NP.

TLR4 in glial cells, a major supportive cell group of CNS, plays a key role in enhancing NP due to systemic inflammatory episodes, such as gut toxicity following chemotherapy or a direct assault on CNS. In addition, glial cells are pivotal for CNS homeostasis, which includes immune surveillance, clearance of debris, regulation of the chemical and ionic composition of the extracellular matrix, and maintenance of the blood–brain barrier integrity.

New data indicate that TLR4 signalling and activation pathways are promoted by the activation of glial cells, such as astrocytes and microglia (Cheng et al., 2015). For instance, glial cells have two distinct states: quiescent basal and activated. Activation of glial cells leads to a proinflammatory response (Lin et al., 2020). Given the role of enterocyte-expressed TLR4 in gut inflammation, it is conceivable that activation of glial cell-expressed TLR4 initiates the induction of a “cytokine storm” in the CNS and provokes NP. In this context, Ramakrishna et al. provide compelling evidence that spinal microgliosis is causally implicated in chemotherapeutics-Paclitaxel-induced NP (Ramakrishna et al., 2019).

#### Gut microbiota, hydrogen-rich water therapy, and NP

Molecular hydrogen (H2) contains antioxidant, anti-inflammatory, and anti-apoptotic properties. Consuming hydrogen-rich water may impact the quality and quantity of gut bacteria (Ge et al., 2017). In their rat-model experiment, Lian et al. (2021) demonstrated that hydrogen-rich water affects the LPS-TLR4 pathway by modifying the diversity of the gut microbiota, thereby reversing the adverse effect of inflammatory cytokines and oxidative stress and alleviating CINP.

#### TRP channels and therapeutic probiotics in NP

Another effect of LPS on gut microbiota mentioned above is that they induce nociceptive neuron depolarization via the activation of transient receptor potential (TRP) channels. The latter is a family of ion channels extensively expressed in primary afferent nociceptors in the dorsal root ganglion. TRP channels function as sensors that convert mechanical, chemical, and thermal stimuli to an electrical current. It is now known that LPS evokes nociceptive neuron depolarization and firing via the activation of TRP channel responses through TLR4 responses (Boonen et al., 2018). Moreover, polyunsaturated fatty acids and intestinal microbial metabolites also lead to peripheral hypersensitivity after TRP channel-TRPV4 activation (Michalak et al., 2016).

Castelli et al. (2018), employing an in vitro model of a sensitive neuron, found that a high-concentration probiotic (De Simone Formulation -DSF formulation) containing some 450 billion bacteria per sachet could reduce CINP by neutralising Paclitaxel-induced increased TRPV1 and TRPV4 channel activation. In clinical terms, such probiotic formulations may serve as effective adjuvant therapy for cold hypersensitivity caused by nerve injury or chemotherapy.

#### CD28 super agonist: a Tregs – population expander therapy in NP

Gut mucosa harbours a dynamic population of Foxp3+ regulatory T cells (Tregs) (Harrison and Powrie, 2013). These cells are indispensable in suppressing the inflammatory response elicited by the activity of commensal gut microbiota. The latter, on the other hand, plays a role in modulating Treg differentiation and its homeostasis. Furthermore, this subpopulation of T lymphocytes can suppress the responses of the adaptive immune system and temper antigen-presenting cell activation by direct interaction and the secretion of anti-inflammatory molecules, including IL-10 and TGF-β (Harrison and Powrie, 2013). In this manner, increasing the number of Tregs reduces neuroinflammation and alleviates mechanical pain hypersensitivity while depleting Tregs potentiates pain in animal models of neuropathy.

For instance, Austin et al. (2012) employed a CD28 super-agonist (CD28SupA), a Treg population expander, to reduce NP in an animal CCI model. Furthermore, in another experiment depleting Tregs with a CD25 antibody in mice with partial sciatic nerve ligation increased mechanical pain sensitivity implying that Tregs help neuropathy-induced pain resolution and their role in neuroimmune crosstalk (Austin et al., 2012). To conclude, Treg activity, modulated by gut microbiota, affects NP indicating a clear axis of microbiota-neuroimmune crosstalk.

## Review limitations

This review provides a comprehensive account of the current data on experimental animal models extant on gut–brain crosstalk and therapeutic targets related to NP. Clinical trials in this context were sparse, and we noted only a single human trial in HIV-infected individuals that evaluated the effect of an antiretroviral on NP. Nevertheless, animal models have thus far helped us to understand the basic neuropathological, biochemical, immunological, and physiologic processes underlying the discussed phenomena.

## Conclusions

We review here, for the first time, the gut microbiome-immune-NP axis and the potential therapeutic targets for alleviating NP. The gut–brain axis is essentially a bidirectional nexus between the central nervous system (CNS) and the gut microbiota. Insults to the neural tissues that evoke an inflammatory response of the CNS, and the consequent NP, are basically mediated through microglial activation, production of inflammatory molecules, and migration of peripheral immune cells into the brain. These lead to the development of a cerebral inflammatory environment that impacts healthy neuronal functionality, eventually precipitating NP. Noteworthy, in this context, are the gut pathologies with increased intestinal barrier permeability and the translocation of microbial and immunologic end products that may enhance cytokine production resulting in NP.

On the other hand, the gut microbiome is a complex and dynamic ecosystem essential for maintaining intestinal homeostasis. Any dysbiosis in the gut microbiota may lead to inflammatory conditions that adversely affect the homeostasis of the neuronal system and thus evoking pain. Particularly noteworthy are the downstream immune pathways and the associated chemicals that are known to evoke pain and hence could be used as potential therapeutic targets in modulating NP.

The discovery of novel therapies and treatments for ailments requires translating ideas from animal models to humans. It necessitates a thorough comprehension of the limits of animal models as well as a careful evaluation of the variations between animal and human biology, genetics, and therapeutic response. Moreover, to ascertain safety and efficacy in treating human diseases, it is crucial to corroborate the findings from animal studies through clinical trials in humans. Clinical trials aid in identifying variations in how humans and animals react to a given treatment and clarify the shortcomings and restrictions of the animal models. Hence, future studies should include clinical trials to further comprehend the role of gut microbiota in NP in order to find new therapeutic targets.

## Data Availability

The accepted manuscript is a systematic scoping review that includes collated data presented in tabular form in the manuscript. The prescribed methodologies for conducting a systematic scoping review were meticulously adhered to and explicitly acknowledged in the Methodology section of the manuscript.
